# Myostatin: A Skeletal Muscle Chalone

**DOI:** 10.1146/annurev-physiol-012422-112116

**Published:** 2022-10-20

**Authors:** Se-Jin Lee

**Affiliations:** 1 Department of Genetics and Genome Sciences, University of Connecticut School of Medicine, Farmington, Connecticut, USA; 2 The Jackson Laboratory for Genomic Medicine, Farmington, Connecticut, USA

**Keywords:** GDF-8, transforming growth factor-β, activin, latency, growth, tissue size

## Abstract

Myostatin (GDF-8) was discovered 25 years ago as a new transforming growth factor-β family member that acts as a master regulator of skeletal muscle mass. Myostatin is made by skeletal myofibers, circulates in the blood, and acts back on myofibers to limit growth. Myostatin appears to have all of the salient properties of a chalone, which is a term proposed over a half century ago to describe hypothetical circulating, tissue-specific growth inhibitors that control tissue size. The elucidation of the molecular, cellular, and physiological mechanisms underlying myostatin activity suggests that myostatin functions as a negative feedback regulator of muscle mass and raises the question as to whether this type of chalone mechanism is unique to skeletal muscle or whether it also operates in other tissues.

## THE CHALONE HYPOTHESIS FOR THE REGULATION OF TISSUE MASS

Substances of the kind under discussion do not fall within the usual definition of a hormone as a systemically distributed chemical substance ‘produced by one tissue with the primary function of exerting a specific effect of functional value on another tissue’ (Huxley 1935) (1). On the contrary, these are substances each of which is produced by a tissue with the primary function of controlling the growth, and perhaps the differentiation, of that same tissue…. It is suggested that the members of this complex of chemical messengers, which may possibly prove to be a family of proteins, should be called chalones.

(2, p. 332)

Over the past several decades, remarkable progress has been made in terms of understanding the molecular and cellular mechanisms underlying cell differentiation events that lead to the formation of individual tissues and organs and the establishment of the basic body plan. Despite this progress, however, very little is understood regarding the control of tissue size. Specifically, relatively little is known as to how tissues and organs grow to their appropriate size during development and how their sizes are then maintained throughout the life of the animal. The control of tissue size is an important topic not only for developmental biology and physiology but also for regenerative medicine, as insights into these regulatory mechanisms could potentially inform the development of strategies to restore tissues damaged by injury or disease.

The control of tissue size has been studied most extensively in those tissues that are capable of regenerating during adult life, and the focus of many of these studies has been the liver, which has an enormous capacity to regrow following loss or injury. The most dramatic experimental illustration of the liver’s regenerative capacity is the partial hepatectomy model in rodents. In mice, surgical removal of approximately two-thirds of the animal’s liver mass triggers a growth response in the tissue remnant, leading to the complete restoration of liver mass over the course of about 10 days. Although the partial hepatectomy model has been used extensively to study the molecular and cellular events occurring during liver growth, a fundamental unanswered question with respect to control of liver size is how the animal “knows” at any given time exactly how much liver mass it has. Specifically, how does the animal know immediately following surgery that it has lost two-thirds of its liver mass and needs to start regenerating? And how does the animal know after approximately 10 days that it has restored its liver back to its original size and that it needs to stop regenerating?

One model proposed over a half century ago to explain this phenomenon was that the entire process might be controlled by a negative growth regulator, which Bullough dubbed a chalone (2). According to this model (2, 3), liver cells produce a liver-specific chalone that circulates in the blood and signals back to hepatocytes to inhibit proliferation. When an animal undergoes a partial hepatectomy, the loss of cells producing the liver chalone would lead to lower circulating levels of this growth inhibitor, specifically to a level below that necessary to maintain hepatocytes in growth arrest. That would then be the trigger to release hepatocytes from growth arrest, leading to liver growth. As the liver grows, the increased liver mass would lead to increased circulating levels of the liver chalone until the liver attains its original size, at which point the chalone would be restored to normal levels sufficient to inhibit further growth. Bullough further suggested that this type of negative feedback regulation might also be involved in the homeostatic control of other tissues as well, with each tissue producing its own specific chalone. Despite intensive efforts in the ensuing years to identify these molecules, the chalone hypothesis was largely abandoned for lack of experimental support. It is now clear that direct, negative feedback regulation of tissue growth by circulating signaling molecules does play an important role in at least one tissue, namely, skeletal muscle. This suggests that dismissal of the chalone hypothesis to explain the control of tissue size may have been premature, at least in the case of skeletal muscle, and raises the question as to whether this type of negative feedback regulation may also be involved in controlling tissue size more generally.

## DISCOVERY OF MYOSTATIN AS A NEGATIVE REGULATOR OF MUSCLE MASS

Myostatin (MSTN) was discovered 25 years ago in a search for new members of the transforming growth factor-beta (TGF-β) superfamily of secreted signaling molecules (4). Like other TGF-β family members, MSTN is made as a precursor protein that undergoes processing by furin proteases to generate an N-terminal propeptide and a C-terminal peptide, a disulfide-linked dimer of which is the actual signaling molecule. It is in this C-terminal region that MSTN shows amino acid sequence similarity to other TGF-β family members, and MSTN forms its own subfamily within the larger superfamily along with the highly related protein growth differentiation factor (GDF)-11, which was originally identified using *Mstn* as a probe (4-7). The sequence of MSTN has been highly conserved through evolutionary selection, with the amino acid sequences of mature MSTN being identical in species as divergent as humans and turkeys (8).

*Mstn* was found to be expressed specifically in the skeletal muscle lineage both during embryonic development and in adult mice (4). In situ hybridization analysis showed that *Mstn* begins to be expressed in midgestation mouse embryos in developing somites, specifically in the myotome compartment that gives rise to skeletal muscle, and continues to be expressed in developing muscle throughout embryogenesis. In adult mice, *Mstn* RNA is expressed almost exclusively in skeletal muscle. Although the tissue specificity of *Mstn* expression is not absolute, *Mstn* RNA is present at readily detectable levels in all skeletal muscles that have been examined and only at very low levels in certain nonmuscle tissues. Within skeletal muscle, the myofibers themselves are the predominant, if not the sole, source of MSTN, as targeting a *Mstn^flox^* allele (9) using a myosin light chain promoter/enhancer-driven cre transgene (*Myl1-cre*), which is expressed by myofibers but not satellite cells (10), can virtually eliminate *Mstn* RNA in whole muscle tissue (11).

The function of MSTN was revealed by gene targeting studies in mice, in which *Mstn*^−/−^ mice were found to have an approximate doubling of skeletal muscle mass throughout the body, demonstrating that MSTN normally acts to limit muscle mass (4). Subsequent genetic studies have shown that the function of MSTN as a negative regulator of muscle mass has been highly conserved through evolution. Naturally occurring *MSTN* mutations having been identified in heavily muscled breeds of cattle (8, 12, 13), sheep (14), and dogs (15), as well as in a hypermuscular human (16), and certain *MSTN* alleles have been shown to correlate with racing performance in whippet dogs (15) and Thoroughbred horses (17-21). Moreover, engineered *MSTN* mutations have been shown to cause increased muscling in rabbits (22), rats (23), swine (24), and goats (25) as well as in certain nonmammalian animals, including zebrafish (26, 27), catfish (28), quail (29), and chickens (30).

Analysis of the phenotype of *Mstn*^−/−^ mice suggested that MSTN plays two distinct roles in regulating muscle mass. Part of the increased muscle mass in these mice results from muscle fiber hyperplasia, implying that MSTN plays a key developmental role in regulating the number of muscle fibers that are formed (4). In this respect, numerous studies have documented that MSTN is capable of signaling directly to myoblasts in culture to regulate cell proliferation and differentiation, consistent with a direct role that MSTN may play in regulating myogenesis in vivo (for reviews, see 31-33). MSTN also appears to have a developmental role in regulating muscle fiber type composition, as muscles of *Mstn*^−/−^ mice exhibit a shift toward more glycolytic fibers as a result of increases primarily in the number of type IIb fibers (34). In addition to this developmental role of MSTN in regulating muscle fiber number and composition, MSTN also regulates the growth of muscle fibers, and it is the combination of increased fiber numbers and increased fiber sizes that accounts for the approximate overall doubling of muscle mass seen in *Mstn*^*−/−*^ mice (4). A hypermuscular phenotype is also seen in *Mstn^flox/flox^*, *Myl1-cre* mice, demonstrating that it is loss of myofiber-derived MSTN that is responsible for the increased muscling, at least with respect to muscle fiber hypertrophy (11, 35). The key role that MSTN plays in regulating postnatal growth of myofibers was clearly documented in studies showing that muscle hypertrophy can be induced either by systemic administration of a monoclonal antibody directed against MSTN to adult wild-type (36) and dystrophic (37) mice or by genetically targeting *Mstn* using a tamoxifen-inducible, ubiquitously expressed cre transgene in adult mice (38). Although MSTN seems to preferentially regulate the formation of type IIb fibers during development, inhibition of MSTN postnatally has been shown to induce hypertrophy of both type I and type II fibers (39-41).

Studies targeting MSTN signaling either genetically or pharmacologically in dystrophic mice as well as in mice in which muscle injury has been induced with cardiotoxin suggest that MSTN also plays an important role during muscle regeneration. Moreover, some, though not all, studies have shown that MSTN is capable of signaling to and regulating satellite cells in culture (for reviews, see 32, 33). Although these studies raise the possibility that direct signaling of MSTN to satellite cells may play a role in muscle regeneration, definitive studies targeting MSTN signaling in satellite cells in vivo have not yet been reported. Several lines of evidence, in fact, suggest that satellite cell activation does not seem to be involved in muscle hypertrophy induced by inhibition of MSTN signaling. In particular, the number of myonuclei per fiber was shown to remain unchanged in muscles induced to undergo hypertrophy, implying that muscle growth resulting from MSTN inhibition occurs without recruitment of satellite cells (39, 40, 42). Similarly, analysis of satellite cells following pharmacologic inhibition of MSTN signaling found no effect on the number of satellite cells per myofiber, at least up to 8 weeks following treatment (39, 40, 42). Moreover, direct tracking of satellite cells marked using a satellite-specific *Pax7-creER* knockin allele to activate a LacZ reporter transgene found no evidence of satellite cell fusion to myofibers during the hypertrophic response (10). Consistent with these findings, muscle hypertrophy due to MSTN inhibition is also seen in mice lacking either Pax7 or syndecan4 (10), both of which have been shown to be important for satellite cell function and development (43, 44). Finally, as discussed in detail below, direct targeting of MSTN receptors in myofibers is sufficient to induce muscle hypertrophy, demonstrating that myofibers are direct targets for MSTN signaling in the regulation of muscle growth (10, 11). Taking all of these data together, it seems clear that under normal physiological conditions, MSTN signals directly to myofibers to limit growth and that muscle hypertrophy induced by MSTN inhibition does not require activation of satellite cells and their fusion to growing myofibers. Additional studies will be required to determine definitively what role, if any, MSTN signaling to satellite cells may play during muscle regeneration.

## MYOSTATIN CIRCULATES IN THE BLOOD

**Figure 1:**
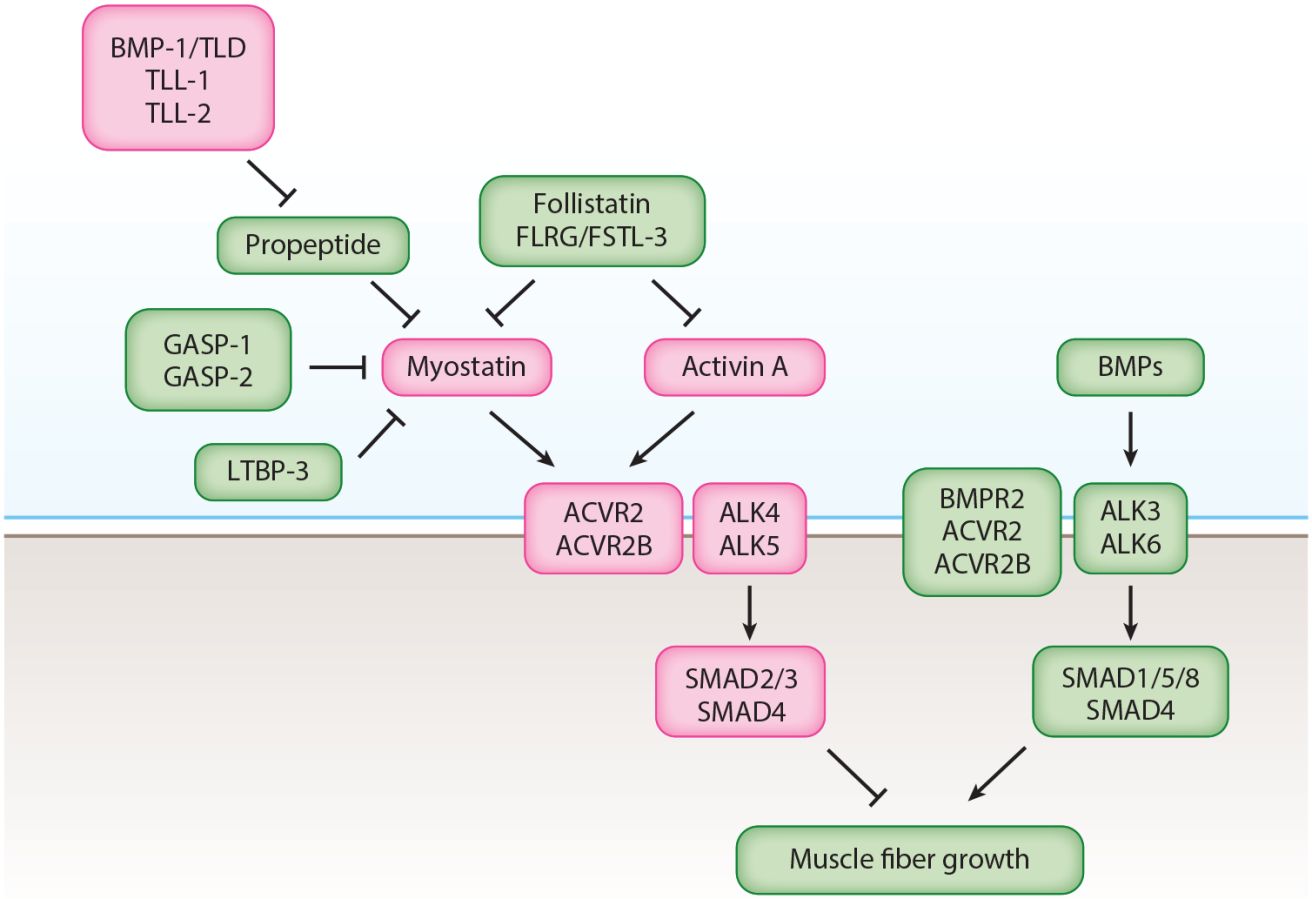
Regulation of muscle mass by myostatin (MSTN), activin A, and bone morphogenetic proteins (BMPs). Following proteolytic processing of the precursor protein, MSTN remains noncovalently bound to its N-terminal propeptide, which maintains MSTN in an inactive, latent state. The latent MSTN complex is activated by members of the BMP-1 family of metalloproteases: BMP-1, TLD (tolloid), TLL-1 (tolloid-like 1), and TLL-2. MSTN is also regulated extracellularly by other inhibitory binding proteins, including GASP-1 (growth and differentiation factor–associated serum protein-1), GASP-2, follistatin (FST), and FSTL-3 (follistatin-like 3) (also called FLRG or follistatin-related gene). Follistatin and FSTL-3 are also capable of binding and inhibiting activin A. LTBP-3 (latent TGF-β binding protein-3) may play a role in regulating the processing of the MSTN precursor protein. MSTN and activin A signal by binding initially to the activin type 2 receptors, ACVR2 (activin receptor type 2) and ACVR2B, which leads to engagement of the type 1 receptors, ALK4 (activin receptor–like kinase-4) and ALK5. ALK4 and ALK5 phosphorylate and activate the SMAD (SMA and MAD-related) proteins, SMAD2 and SMAD3, which form a complex with SMAD4 and act to inhibit muscle growth. Although the specific components utilized by BMPs in muscle have not been completely elucidated, BMPs are capable of binding to the type 1 receptors, ALK3 and ALK6, and then engaging the type 2 receptors, BMPR2 (bone morphogenetic protein receptor type 2), ACVR2, and ACVR2B. ALK3 and ALK6 phosphorylate and activate SMAD1, SMAD5, and SMAD8, which form a complex with SMAD4 and act to induce muscle growth. Components shown in green act to induce muscle growth, and components shown in pink act to inhibit muscle growth.

MSTN produced by muscle circulates in the blood (45); in fact, circulating levels of MSTN are quite high, being in the range of 60–80 ng/mL in mice (9) and approximately an order of magnitude lower in humans (46). Although MSTN protein is readily detectable in mouse serum by Western or enzyme-linked immunosorbent assay (ELISA) analysis, MSTN activity in serum, measured using a Smad-responsive luciferase reporter gene assay, could only be detected upon acid treatment, suggesting that most, if not all, of the circulating MSTN protein is present in complexes with inhibitory binding proteins (45) (Figure 1). Direct analysis of MSTN protein complexes in the blood by affinity purification using a monoclonal antibody directed against the C-terminal domain identified several of these binding partners. The major binding protein for mature MSTN identified in the blood was its own propeptide (47), which is the N-terminal fragment generated by proteolytic cleavage of the precursor protein. Prior studies had shown that following proteolytic processing, the propeptide remains noncovalently bound to the MSTN C-terminal dimer and maintains it in an inactive, latent state (48, 49). Based on the molar ratios of the two proteins present in the eluates following affinity purification, it was estimated that this latent complex represents the vast majority of MSTN that circulates in the blood (47). Affinity purification of MSTN complexes from the blood also identified two other proteins bound to mature MSTN in addition to the propeptide. One of these was follistatin-like 3 (FSTL-3, also called FLRG) (47), which is a protein containing follistatin domains and, like follistatin (FST) itself, known to be capable of binding other TGF-β family members as well, most notably activins (50-53). A second protein bound to MSTN in the blood was GASP-1 (growth and differentiation factor–associated serum protein-1) (54), which had previously been identified and named WFIKKN2 as a protein containing a follistatin domain as well as multiple domains associated with protease inhibitors (55). It is certainly possible that additional MSTN complexes present in blood may not have been detected using the affinity purification approach, as certain binding proteins may have shielded the epitope recognized by the anti-MSTN monoclonal antibody.

Myofibers are clearly the predominant source of circulating MSTN protein, as plasma MSTN levels have been shown to be severely reduced in *Mstn^flox/flox^*, *Myl1-cre* mice, in which *Mstn* has been targeted specifically in myofibers (11). In addition to skeletal muscle, two other tissues may contribute to the circulating pool of MSTN protein, at least in certain physiological conditions. One of these tissues is the heart, in which *Mstn* expression is normally very low but is significantly upregulated following heart injury (35, 56-62). In one study, *Mstn* expression was shown to increase in the heart following transverse aortic constriction (TAC) to induce pressure overload heart failure in mice, leading to two- to threefold increases in circulating MSTN levels as well as skeletal muscle atrophy (35). Most importantly, this increase in circulating MSTN following TAC is not seen in mice in which *Mstn* is targeted in cardiomyocytes using an *Nkx2.5-cre* transgene, demonstrating that the heart is the source of this increased circulating protein. Moreover, *Mstn^flox/flox^*, *Nkx2.5-cre* mice are resistant to TAC-induced skeletal muscle atrophy, raising the possibility that cardiomyocyte-derived MSTN may be a key mediator of cardiac cachexia. A second tissue that may contribute to the circulating MSTN pool is brown adipose tissue (BAT). *Mstn* expression in BAT is normally repressed by the transcription factor interferon regulatory factor 4 (IRF-4), and *Mstn* expression in BAT is upregulated in mice in which *Irf4* has been targeted specifically in BAT (63). Moreover, targeting *Irf4* in BAT leads to increases in circulating MSTN levels, which are also seen in mice maintained at thermoneutrality, and conversely, overexpression of *Irf4* in BAT leads to reductions in circulating MSTN levels. Based on these studies, BAT has been suggested to be an important source for circulating MSTN, although unlike the case for heart, definitive studies measuring circulating MSTN levels in mice in which *Mstn* has been targeted in BAT have not yet been reported.

Because MSTN protein can be readily detected in the blood, numerous studies have measured levels of circulating MSTN in both mice and humans in a variety of physiological and disease states. A key question in this regard has been the biological significance of circulating MSTN protein levels, specifically in terms of whether MSTN levels in the blood are simply a reflection of physiological changes occurring in muscle, including changes in overall muscle mass, and/or whether changes in circulating MSTN levels translate into changes in levels of MSTN signaling activity in muscle. Overexpression studies in mice have shown that MSTN can act systemically to regulate tissues distant from its site of synthesis. In particular, nude mice injected intramuscularly at a single site with Chinese hamster ovary (CHO) cells producing high levels of native MSTN protein were shown to develop a cachexia-like syndrome characterized by rapid loss of both muscle and fat throughout the body (45). Although these results clearly demonstrate that MSTN is capable of acting in a systemic manner, the levels of circulating MSTN protein in these mice were supraphysiologic, reaching levels more than an order of magnitude higher than those seen in normal mice; in fact, even though the CHO cells were engineered to produce a full-length MSTN precursor protein, including the propeptide, biologically active MSTN protein could be detected in the blood even in the absence of acid activation, suggesting that the concentrations of inhibitory binding proteins were insufficient to maintain the excess MSTN in an inactive state.

Although additional studies will be required to definitively determine whether MSTN truly acts as a hormone under normal physiological conditions, three lines of genetic evidence suggest that circulating MSTN protein can enter the active pool and regulate muscle growth. First, as discussed above, targeting *Mstn* in the heart can prevent skeletal muscle atrophy following heart injury, consistent with circulating MSTN produced by the heart being capable of signaling to skeletal muscle (35). The second line of evidence is the observation that the *Mstn* loss-of-function mutation exerts a maternal effect on muscle size (64). In particular, *Mstn* exhibits a dose-dependent effect on muscle mass, with *Mstn*^+/−^ mice having an increase in muscle weights of approximately 20–25% compared to the approximate doubling seen in *Mstn*^−/−^ mice (4), but the effect of heterozygous loss of *Mstn* was found to differ depending on the parent-of-origin of the mutant allele; that is, *Mstn*^+/−^ mice exhibit greater increases in muscle mass when the *Mstn* deletion allele is transmitted through the mother compared to when it is transmitted through the father (64). This difference is also observed in *Mstn*^−/−^ mice depending on whether the mother is homozygous or heterozygous for the mutation, ruling out imprinting of the *Mstn* gene as a possible explanation. Moreover, this maternal effect is maintained even when newborn mice are transferred to mothers of different genotypes immediately after birth, ruling out the possibility that these differences in muscle mass are effects occurring during postnatal mothering, such as transfer of MSTN from the mother to the offspring through nursing. Hence, the maternal effect is likely to result from the transfer of key molecules from the mother to the fetus across the placenta, and the simplest possibility is that the key molecule is MSTN itself. A third line of evidence comes from “single animal parabiosis” studies in which the *Mstn* gene was targeted only in the posterior half of mice using a *Cdx2-cre* transgene (9), which is expressed in all cells posterior to the umbilicus but not in cells anterior to the umbilicus (65). Examination of muscles located in the posterior versus anterior regions of *Mstn^flox/flox^*, *Cdx2-cre* mice provided evidence for both local and systemic effects of MSTN (9). Specifically, muscles in the posterior region of these mice showed dramatic increases in weights compared to muscles in the anterior region, consistent with MSTN having an autocrine/paracrine mode of action. Detailed examination of these muscles, however, revealed that the effects in the posterior region were smaller than might be expected for the complete loss of MSTN, likely reflecting rescue by circulating MSTN protein made by anterior muscles, and that the anterior muscles exhibited some hypertrophy, likely reflecting the lower circulating levels resulting from the loss of MSTN production by the posterior muscles. All of these data are consistent with circulating MSTN being capable of entering the active pool and MSTN having both autocrine/paracrine and endocrine modes of action.

## MYOSTATIN RECEPTORS

Given that MSTN circulates in the blood and may have an endocrine mode of action, how is specificity achieved in terms of its effects on skeletal muscle? One possibility could be that signaling is restricted to skeletal muscle as a result of tissue-specific expression of key receptor components (Figure 1). Like other TGF-β family members, MSTN signals through a combination of type 2 and type 1 receptors. Initial cross-linking studies showed that MSTN is capable of binding directly to the two activin type 2 receptors, ACVR2 and ACVR2B (also called ActRIIA and ActRIIB) (48), and the key role that these receptors play in regulating MSTN activity and muscle growth in vivo has been documented by genetic studies in mice. In particular, transgenic overexpression of a dominant negative form of ACVR2B, namely, a truncated receptor lacking the cytoplasmic kinase domain, under the control of a skeletal muscle–specific *Myl1* promoter/enhancer was shown to cause a hypermuscling phenotype characterized by increased fiber numbers and increased fiber sizes (48). Moreover, increased muscling is also seen in mice in which *Acvr2* and/or *Acvr2b* have been genetically targeted either globally (66) or just in myofibers (10, 11). The two activin type 2 receptors are partially functionally redundant with each other, as targeting *Acvr2* and *Acvr2b* simultaneously in myofibers leads to a much more substantial effect than targeting either alone (11). Pharmacological studies have also documented the key role that these receptors play in regulating muscle growth postnatally. Specifically, either a decoy form of ACVR2B consisting of its ligand-binding domain fused to an immunoglobulin Fc domain (66) or a monoclonal antibody directed against the activin type 2 receptors (67-69) is capable of inducing muscle fiber hypertrophy when administered systemically to adult mice. At high doses, in fact, just two injections of the ACVR2B/Fc decoy receptor can induce over 50% muscle growth over a span of two weeks (66). Binding of MSTN to the activin type 2 receptors then engages the type 1 receptors, ALK4 and ALK5 (70). Targeting either *Alk4* or *Alk5* in myofibers has a small effect in terms of increased muscling, but targeting *Alk4* and *Alk5* simultaneously leads to massive increases in muscle mass (11). Hence, as in the case of the type 2 receptors, the type 1 receptors are also partially functionally redundant with one another in muscle.

The increased muscling seen upon targeting these receptors is consistent with their roles in regulating MSTN signaling, but it is clear that other TGF-β family members also play important roles in regulating muscle mass. In particular, the ACVR2B/Fc decoy receptor is capable of inducing considerable muscle growth when administered systemically not only to wild-type mice but also to *Mstn*^−/−^ mice, implying that at least one other TGF-β family member capable of binding ACVR2B must also function to limit muscle growth (66). Moreover, like MSTN, this other TGF-β family member likely signals through ALK4 and/or ALK5, as targeting these two type 1 receptors together leads to more substantial increases in muscle mass than targeting just MSTN (11). Two lines of evidence have identified activin A, which is a dimer of inhibin βA subunits, as a key ligand that cooperates with MSTN in maintaining muscle homeostasis. One line of evidence comes from genetic studies targeting *Inhba*, which encodes the βA subunit. Although homozygous loss of *Inhba* leads to embryonic lethality (71), heterozygous loss of *Inhba* was found to result in small but significant increases in muscle mass in adult mice (72). Moreover, simultaneously targeting *Inhba* and *Mstn* in a regionally restricted manner, specifically in the posterior region of mice using a *Cdx2-cre* transgene, was shown to cause more substantial increases in muscle mass than targeting *Mstn* alone, documenting that the two ligands are partially functionally redundant (73). A second line of evidence comes from studies targeting activin A using specific inhibitors. One set of studies used an adeno-associated virus (AAV) vector to deliver a modified form of the activin A propeptide locally to the tibialis anterior muscle (74). This modified activin A propeptide, which is a specific inhibitor of activin A (75), was shown to induce muscle hypertrophy on its own but to have a more substantial, synergistic effect upon codelivery of an AAV vector expressing the MSTN propeptide (74), which is a specific inhibitor of MSTN and GDF-11 (48, 49). Another set of studies examined the effect of blocking these ligands by systemic administration of monoclonal antibodies directed against either MSTN (REGN1033) or activin A (REGN2477) (76). As has been shown with other MSTN-neutralizing monoclonal antibodies, treatment of mice with REGN1033 induces muscle hypertrophy, and although blocking activin A with REGN2477 has a minimal effect when administered alone, REGN2477 greatly potentiates the anabolic effect of REGN1033 on muscle when they are coadministered. Taking all of these studies together, it seems clear that activin A is at least one key ligand that cooperates with MSTN to limit muscle growth.

Given that both MSTN and activin A play important roles in regulating muscle growth and that both are capable of utilizing the same receptors for signaling in vitro, a key question is whether each ligand utilizes each of these receptors in vivo. In this regard, targeting *Mstn* alone has been shown to have a greater effect on muscle mass than targeting any of the receptor components individually. This implies that MSTN almost certainly utilizes both type 2 receptors, ACVR2 and ACVR2B, and both type 1 receptors, ALK4 and ALK5, for signaling in muscle (11). Moreover, studies genetically targeting each of these receptor components in combination with *Mstn* have suggested that all four receptors are also likely utilized by activin A, with perhaps ALK5 playing a lesser role than ALK4 in mediating activin A signaling. A related question is whether these ligands utilize distinct combinations of type 2 and type 1 receptors in vivo. One approach to elucidating the roles of specific type 2–type 1 receptor combinations utilized in vivo has been to examine the effect of simultaneously targeting one type 2 receptor along with one type 1 receptor (11). For example, studies simultaneously targeting *Acvr2b* along with *Alk4* showed that the one remaining combination of ACVR2 with ALK5 is sufficient to maintain muscle mass at close to normal levels. Targeting all possible combinations in this manner showed that ALK4 plays a lesser role in general than ALK5, with perhaps the ACVR2B/ALK4 combination playing the least important role, but that all four possible receptor combinations seem to be utilized in vivo. Hence, although these findings suggest that neither ligand signals exclusively through just a single type 2–type 1 receptor combination, these data are consistent with certain receptor combinations perhaps being used preferentially by certain ligands.

The interpretation of these genetic studies is further complicated by the discrepancy that was observed upon targeting the two type 2 receptors versus targeting the two type 1 receptors (11). In particular, simultaneously targeting *Alk4/Alk5* in myofibers had a much more substantial effect on muscle mass than simultaneously targeting *Acvr2/Acvr2b*. One possible explanation for this discrepancy could be that targeting *Alk4/Alk5* may disrupt signaling by MSTN, activin A, or other growth-suppressing ligands that are capable of utilizing other type 2 receptors besides ACVR2 and ACVR2B. Three other type 2 receptors for the TGF-β family of ligands have been identified, namely, Mullerian inhibiting substance receptor type 2 (MISR2), bone morphogenetic protein receptor type 2 (BMPR2), and TGF-β receptor type 2 (TGFBR2) (for a review, see 77). MISR2 is unlikely to play a role, at least in mediating the effects of MSTN and/or activin A, as MISR2 is highly specific for anti-Mullerian hormone. Based on binding specificity, BMPR2 is also unlikely to play a role, and moreover, targeting *Bmpr2* simultaneously along with *Acvr2/Acvr2b* was shown to have no added effect on muscle mass compared to targeting just *Acvr2/Acvr2b* (11). That leaves TGFBR2 as the only remaining possible candidate among the known type 2 receptors. TGFBR2 is highly specific for the TGF-β isoforms, and TGF-β binding to TGFBR2 then leads to coupling with ALK5 for signaling (for a review, see 77). Hence, a possible explanation for the observation that targeting *Alk4/Alk5* gives a greater effect than targeting *Acvr2/Acvr2b* could be that TGF-β itself cooperates with MSTN and activin A to limit muscle growth, in which case targeting *Alk5* would have the added effect of blocking TGF-β signaling through TGFBR2.

An alternative explanation for this discrepancy could be that targeting the two activin type 2 receptors might eliminate signaling by ligands that normally act antagonistically to MSTN and activin A but utilize other type 1 receptors besides ALK4 and ALK5. The most likely possibility is that targeting *Acvr2/Acvr2b* might block signaling by certain BMPs, which appear to counteract signaling by MSTN and activin A in muscle (Figure 1). In particular, activation of BMP signaling by overexpression of either BMP-7 or a constitutively active BMP type 1 receptor (ALK3) has been shown to induce muscle hypertrophy (78, 79). Conversely, inhibition of BMP signaling by overexpression of either noggin, which binds and inhibits BMPs (80), or Smad6, which inhibits BMP-induced Smad1/5/8 activity (81), has been shown to induce muscle atrophy (78, 79). The identities of the endogenous BMP ligands that are critical in regulating muscle homeostasis under normal physiological conditions are not entirely clear, but expression of genes encoding two BMP-related ligands, GDF-5 and GDF-6, is upregulated in denervated muscle (78, 79), and *Gdf5*^−/−^ mice exhibit more muscle loss than wild-type mice following denervation (79). Moreover, activation of BMP signaling is seen in muscle hypertrophy resulting from the inhibition of MSTN and activin A signaling. Specifically, overexpression of FST, which is a potent MSTN and activin A inhibitor that can induce dramatic muscle hypertrophy (48, 64), leads to increased levels of phospho-Smad1/5 (78).

All of these findings suggest that muscle mass may be controlled by the relative activities of MSTN/activin A versus BMPs, which are known to activate different groups of receptor-regulated Smads (R-Smads) that mediate signaling. According to one model (79), phospho-Smad2/3, whose levels are increased upon MSTN/activin A signaling, and phospho-Smad1/5/8, whose levels are increased upon BMP signaling, would compete for limiting amounts of Smad4, and the relative amounts of these R-Smads would thereby determine whether the physiological response is shifted to muscle atrophy versus muscle growth. In this respect, mice lacking Smad4 exhibit slight reductions in muscle mass in normal physiological conditions but dramatic muscle atrophy following denervation (79). The discrepancy seen upon targeting *Acvr2/Acvr2b* versus *Alk4/Alk5* in myofibers, however, suggests that competition for signaling components may occur farther upstream at the level of the receptors. In particular, although BMPs can utilize their own type 2 receptor for signaling, namely, BMPR2, targeting *Bmpr2* in myofibers leads to only relatively small decreases in muscle mass, implying that other type 2 receptors likely mediate BMP signaling in muscle (11). In this respect, BMPs are also capable of utilizing activin type 2 receptors (for a review, see 77), raising the possibility that targeting *Acvr2/Acvr2b* in myofibers could potentially block signaling not only by MSTN and activin A but also by BMPs, which would lead to counteracting effects. In contrast, because BMPs utilize distinct type 1 receptors, namely, ALK3 and ALK6, targeting *Alk4/Alk5* would block signaling by MSTN and activin A but not affect signaling by BMPs, thereby leading to a much more robust hypertrophy effect.

In addition to the type 2 and type 1 receptors, one other receptor component has been implicated as playing a role in MSTN signaling. In particular, one study showed that Cripto (*Cfc1b*), which has been shown to act as a coreceptor for certain ligands and as an antagonist for other ligands (for reviews, see 82, 83), is required for MSTN signaling through ALK4 in cultured C2C12 myoblasts (84). Targeting *Cfc1b* in myofibers, however, either alone or in combination with each of the type 2 and type 1 receptors, has no effect on muscle mass (11). Most significantly, targeting *Cfc1b* simultaneously with *Alk5* has no added effect compared to targeting *Alk5* alone, implying that signaling through ALK4 does not require Cripto, at least in myofibers. One caveat in interpreting the results of these studies is that Cripto has been shown to have the opposite effect with respect to activin A, namely, to inhibit signaling (85). Hence, it is formally possible that targeting *Cfc1b* in myofibers leads to inhibition of MSTN signaling but enhancement of activin A signaling, with these effects offsetting one another and resulting in minimal net changes in overall combined signaling activity. A second caveat is that Cripto appears to have both cell-autonomous and cell-nonautonomous modes of action (for a review, see 83), so Cripto made by other cell types could potentially influence signaling to myofibers. In this respect, Cripto expressed by satellite cells has been shown to play a role in regulating muscle regeneration and, in this case, Cripto has been suggested to antagonize MSTN signaling (86).

Based on the receptor components that have been identified to date, there is no clear model that can adequately explain how these components could be deployed to achieve specificity of MSTN signaling for muscle. In the case of the type 2 and type 1 receptors, preferential utilization of certain combinations of ACVR2 and/or ACVR2B with ALK4 and/or ALK5 by MSTN has not been entirely ruled out, but each of these receptors appears to play at least some role in mediating MSTN signaling, and these same receptor components are also utilized by multiple other members of the TGF-β family of ligands in regulating multiple tissues throughout the body. It is also possible that other, as yet unidentified components may be involved in conferring some degree of specificity, either by serving as coreceptors that are essential for MSTN signaling to myofibers or by acting as antagonists to prevent MSTN signaling to other cell types. Hence, although considerable progress has been made in terms of elucidating the components of this signaling system, additional work will be required to investigate the possibility that other components may also exist and to unravel the complexity by which these components are utilized by different ligands in muscle. This will be potentially important not only for understanding tissue specificity but also for developing strategies to optimally target this pathway for clinical applications.

## EXTRACELLULAR REGULATION OF MYOSTATIN

An alternative mechanism for achieving specificity could be modulation of MSTN activity by extracellular binding proteins and, indeed, a number of inhibitory binding proteins have been identified for MSTN (Figure 1). Without question, a key binding protein for MSTN is its own propeptide. As discussed earlier, the N-terminal propeptide remains noncovalently bound to the mature C-terminal dimer following proteolytic processing of the MSTN precursor protein (48). The propeptide maintains MSTN in an inactive, latent state (48, 49), and the latent complex of MSTN with its propeptide is the major circulating form of MSTN (47). This latent complex can be activated artificially in vitro by either acid or heat treatment to dissociate the propeptide (45, 87). Biochemical studies have demonstrated that latent MSTN can also be activated by cleavage of the propeptide at aspartate 76 by members of the BMP-1/tolloid family of metalloproteases, which includes four proteases encoded by three genes: *Bmp1, Tll1*, and *Tll2* (87). The key role that this mechanism plays in vivo is supported by genetic studies in which a point mutation changing aspartate 76 to alanine, which renders the propeptide resistant to cleavage by BMP-1/tolloid proteases in vitro (87), was introduced into the germline of mice (88). Mice homozygous for the D76A point mutation were shown to have circulating levels of MSTN that are increased by over tenfold and yet exhibit a hypermuscling phenotype with muscle weights approaching those seen in mice completely lacking MSTN, reflecting the inability of latent MSTN to be activated in *MSTN^D76A/D76A^* mice. In addition, mice carrying a loss-of-function mutation in the gene encoding one of these proteases, TLL-2, also exhibit increases in muscle mass, although the relatively small magnitude of these increases suggests that there is likely functional redundancy among these proteases (88), which are each capable of cleaving the propeptide and activating the latent complex in vitro (87). Given that the vast majority of circulating MSTN is bound to its propeptide and that circulating MSTN seems capable of entering the active pool, it is likely that activation of the latent complex by BMP-1/tolloid proteases occurs locally at the site of MSTN action in muscle. Hence, it seems reasonable that local activation of latent MSTN could be one major mechanism for achieving specificity of MSTN action in muscle.

In addition to the propeptide, two other proteins, FSTL-3 and GASP-1, have also been found to be complexed to MSTN in the blood, as discussed earlier. Both of these proteins are capable of inhibiting MSTN activity in vitro (47, 54, 89, 90) and causing increases in muscle mass when overexpressed in mice (64, 91). Genetic studies have shown that mice lacking FSTL-3 have normal muscle mass (72, 92) but that mice lacking GASP-1 and/or GASP-2 (also called WFIKKN1), which is related in sequence to GASP-1 (55,93), have reduced muscle mass, a shift toward oxidative fiber types, and an impairment in muscle regeneration following cardiotoxin injury, all consistent with increased signaling through the MSTN pathway (90). The effects of targeting *Gasp1* and/or *Gasp2* likely result from increased signaling of MSTN itself, as GASP-1 and GASP-2 seem to be specific for MSTN (andGDF-11) and are unable to bind and inhibit activin A (54, 90). Compared to what is known about the role of the propeptide, however, very little is understood regarding the precise roles of these other inhibitory binding proteins in modulating MSTN activity in vivo. In the case of the propeptide, the latent complex of propeptide and mature MSTN forms in muscle directly as a result of processing of the precursor protein and is likely activated by proteolytic cleavage of the propeptide by BMP-1/tolloid proteases at the target site. In the case of the other binding proteins, it is not known when or where complexes with MSTN form, nor is it known what the key tissue sources for these binding proteins might be with respect to regulation of MSTN signaling. An intriguing possibility is that MSTN produced by muscle may form complexes with inhibitory proteins made by other tissues, which would be one potential mechanism for cross talk between muscle and other tissues. Finally, it is not known whether these complexes can even be activated in vivo, let alone the molecular mechanisms that may be involved.

Another MSTN binding protein is FST, although unlike the propeptide, FSTL-3, and GASP-1, FST was not one of the proteins detected by affinity purification of MSTN complexes from the blood. FST was originally identified for its ability to inhibit secretion of follicle stimulating hormone by pituitary cells (94) and subsequently shown to act by binding and inhibiting activins (95). FST was later shown to be capable of binding other TGF-β family members as well, including BMPs (96-98), GDF-11 (5), and MSTN (48). FST is a potent inhibitor of MSTN in vitro in receptor-binding assays, and transgenic overexpression of FST in skeletal muscle can cause dramatic muscle growth, mostly due to muscle fiber hypertrophy (48, 64). The hypermuscling phenotype seen upon overexpression of FST seems to result from inhibition of not only MSTN but also activin A, as overexpression of FST in *Mstn*^−/−^ mice can cause yet another doubling of muscle mass, leading to mice with an overall quadrupling of muscle mass (64), similar to the extreme muscling seen in mice in which *Alk4* and *Alk5* have been targeted together in myofibers (11).

The essential role that FST plays in regulating muscle mass in vivo has been documented by genetic studies targeting *Fst* in mice. Although *Fst*^−/−^ mice die immediately after birth, newborn *Fst*^−/−^ mice have reduced muscle tissue (99), consistent with overactivity of MSTN during muscle development. Moreover, even heterozygous loss of *Fst* leads to reduced muscle mass, a shift toward oxidative fiber types, and impaired muscle regeneration in adult mice, which appear to result from the loss of inhibition of both MSTN and activin A (72). More complete loss of FST, which was achieved regionally by targeting *Fst* in all cells in the posterior region of mice using the *Cdx2-cre* transgene, results in much more substantial muscle size reductions and fiber type shifts as well as in intramuscular fat accumulation, which are seen in muscles located in the posterior region but not in muscles located in the anterior region (73). These findings are significant in that FST is known to exist in multiple forms with distinct biodistribution properties. Specifically, the predominant form that circulates in the blood is the full-length FST315 isoform, whereas the truncated FST288 isoform, which lacks the C-terminal portion capable of shielding the heparinbinding domain, remains locally bound to the extracellular matrix (100). The differential effects seen in posterior versus anterior muscles of *Fst^flox/flox^*, *Cdx2-cre* mice suggest that FST acts locally rather than systemically in regulating muscle, implying that FST288 plays a more important role than FST315 in regulating signaling of MSTN and activin A to muscle (73).

The fact that FST seems to act locally rather than systemically to regulate MSTN activity suggests another possible mechanism for achieving tissue specificity. In particular, one could hypothesize that expression of FST in nonmuscle tissues could be one way to inhibit signaling by MSTN protein reaching those tissues via the circulation. This type of mechanism could also be extended to the other MSTN binding proteins, although whether those other proteins act locally or systemically to regulate MSTN has not yet been determined. In the case of FSTL-3 and GASP-1, for example, it remains possible that protein bound to MSTN in the blood may represent terminal complexes that are incapable of being activated. Whatever the roles of each of these proteins may be, the complexity of this regulatory network involving multiple extracellular binding proteins certainly would allow for a variety of mechanisms by which MSTN signaling may be restricted to muscle, with two possible mechanisms being activation of latent MSTN specifically in muscle and inhibition of MSTN by binding proteins in nonmuscle tissues.

## ROLE OF MYOSTATIN IN REGULATING THE OVERALL BALANCE BETWEEN MUSCLE AND FAT

The superimposition of a complex group of inhibitory binding proteins on this signaling network potentially allows MSTN activity not only to be restricted to skeletal muscle but also to be regulated differentially in individual muscles, such as by local control of BMP-1/tolloid proteases or levels of inhibitory binding proteins in response to local stimuli. If MSTN activity is indeed regulated primarily through these types of mechanisms operating locally at the target site, what then is the physiological role of circulating MSTN? Although it is certainly possible that MSTN may signal directly to tissues other than skeletal muscle, one answer to this fundamental question may lie in the physiological consequences of having excess skeletal muscle as a result of loss of MSTN signaling. In particular, loss of MSTN leads to profound changes in body composition in terms of not only increased muscle mass but also reduced body fat, which becomes more prominent as animals age (101). This suppression of fat accumulation can even be seen when *Mstn*^−/−^ mice are placed on high-fat diets (102) or when the *Mstn* null mutation is introduced into the genetically obese strains, *Ob/Ob* and *A^y^* (101). MSTN signaling also affects glucose metabolism, with *Mstn*^−/−^ mice able to maintain normal or lower fasting glucose levels despite having lower insulin levels (11, 101) and loss of MSTN capable of partially suppressing the development of hyperglycemia and enhancing glucose clearance in genetically obese mice (101). Reduced fat accumulation and improved glucose metabolism have also been documented in mice treated with MSTN inhibitors, demonstrating the important metabolic function that MSTN plays in adult mice (103-105), as well as in mice in which the type 2 receptors, ACVR2 and ACVR2B, have been targeted in myofibers (11). Based on this latter finding, it seems clear that the anabolic effects of blocking signaling to muscle are sufficient to generate the beneficial effects on adiposity and glucose metabolism, although the possibility that the loss of signaling to other tissues, such as adipose tissue, may also play a role has not been completely ruled out.

The fact that MSTN can affect body composition with respect to both muscle and fat suggests a plausible explanation for the role of circulating MSTN protein. I have speculated previously that perhaps the MSTN regulatory system has evolved to be so complex because one of the primary physiological functions of MSTN may be to regulate the overall metabolic balance between muscle and fat (106). By having a master regulator of muscle mass circulate in the blood, its circulating levels could potentially be used both as a gauge for how much skeletal muscle mass is present throughout the body and as a mechanism for setting global limits on skeletal muscle mass in different physiological states. At the same time, by superimposing the possibility of local regulation of MSTN signaling at specific sites, this regulatory system could also potentially be used to control sizes of individual muscles independently. The complexity of this regulatory network, with its multiple layers of regulatory components, would therefore serve to regulate the overall balance between muscle and fat throughout the body while at the same time allowing for muscle to respond to local stimuli. For animals in the wild, maintaining the appropriate balance between building muscle and storing fat is, of course, critical for adapting to different physiological states and environment conditions. For humans, however, the MSTN regulatory system could almost be considered as an evolutionary vestige from a time when we had much less control over our environment.

In that sense, we may be able to manipulate this pathway in muscle with relative impunity, which is one feature of this regulatory system that makes it so attractive for drug development. Indeed, over the past couple of decades, there have been extensive efforts by the academic and pharmaceutical communities to develop strategies to target MSTN signaling for clinical applications and, to date, at least nine pharmaceutical and biotechnology companies have tested MSTN inhibitors in clinical trials (Table 1) (for a review, see 107). These inhibitors have included monoclonal antibodies or functional equivalents directed against mature MSTN (34, 108-113), antipropeptide monoclonal antibodies capable of preventing activation of latent MSTN (114-117), a monoclonal antibody directed against the activin type 2 receptors (67-69), a decoy form of the ACVR2B receptor (66, 118), and a biologic based on FST (119). Collectively, these MSTN inhibitors have been tested in patients with muscle loss due to a wide range of conditions, including various forms of muscular dystrophy (120-122), sporadic inclusion body myositis (123-125), spinal muscular atrophy, age-related sarcopenia (126, 127), muscle loss following falls and hip surgery (128, 129), cachexia due to cancer (130), chronic obstructive pulmonary disease (131), and end-stage renal disease. One drug candidate has also been tested in patients with metabolic diseases, specifically obese patients with type 2 diabetes (132).

Although there are some promising drug candidates still being pursued for some clinical indications, no MSTN inhibitor has yet reached drug approval, and the reasons for the failed trials are likely both diverse and complex (for a review, see 107). A major challenge in these trials has been that although all of the drugs are capable of increasing muscle mass, the effects seen in humans have been quite modest compared to those seen in mice using these same inhibitors. In this respect, circulating levels of MSTN in humans are significantly lower than those seen in mice, which has led to the suggestion that perhaps activin A plays a relatively larger role in regulating muscle mass in humans than it does in mice (76). This, however, cannot account for the lower effects seen in humans with the decoy ACVR2B/Fc receptor or monoclonal antibody directed against the activin type 2 receptors, as these drugs are capable of blocking both MSTN and activin A. An alternative possibility is that perhaps the relative balance between MSTN/activin A signaling and BMP signaling may be shifted in humans compared to mice. Whatever the explanation may be, the clinical experience suggests that new strategies may be required to generate more substantial effects on muscle, as it seems clear that the full muscle anabolic potential of targeting this signaling pathway has not yet come close to being fully tapped.

Another major challenge in these trials has been defining clinically meaningful outcome measures, and the modest effects on muscle mass and strength seen with the various drugs in humans have led to inconsistent outcomes in terms of functional improvements. Indeed, it is not clear how much of an increase in muscle mass and strength would be required to achieve meaningful improvements in metrics such as six-minute walk distance, time to rise from a chair, stair climbing time, gait speed, etc., which are typically used in many trials. In this respect, one indication that has received considerable attention recently is spinal muscular atrophy (SMA). Though still unpublished, the biopharmaceutical company Scholar Rock released encouraging data from a phase 2 trial in SMA patients using apitegromab, which is a monoclonal antibody directed against the MSTN propeptide, in combination with a splice modulator to increase survival motor neuron (SMN) protein levels. It is certainly possible that the beneficial effects seen in this trial may reflect the mechanism of action of apitegromab in blocking activation of latent MSTN, which has been suggested to be advantageous to blocking mature MSTN. Another possibility could be that SMA as an indication may be particularly responsive to MSTN inhibition compared to other indications, particularly in the setting in which the underlying disease is being treated with splice modulators. It is also possible, however, that the clinical benefits seen in this trial may simply reflect the battery of tests used to assess function in SMA patients. Specifically, SMA patients are standardly assessed using the Hammersmith scale, which measures performance on 33 individual tasks, with an improvement of 3 points out of a possible 66 points being accepted by clinicians as significant clinical improvement. Hence, the wider window afforded by the multitask set of assessments may provide greater sensitivity in demonstrating functional improvement in response to MSTN inhibition. At least some of these questions should be answered in the near future, as three companies—Scholar Rock, Roche/Chugai, and Biohaven—are each currently launching phase 3 trials with their respective MSTN inhibitors in patients with SMA.

Finally, although the results of many of these trials have been somewhat disappointing in terms of the magnitude of muscle mass increases and functional improvements, one of the striking, consistent findings in these trials and other clinical studies has been the beneficial effects observed in terms of fat reduction and/or glucose metabolism (125-129, 131-137). The most compelling data in this regard were reported by Novartis in their trial of bimagrumab, which targets the activin type 2 receptors, in obese patients with type 2 diabetes. In this trial, treatment with bimagrumab resulted in a 4.4% increase in lean body mass, a 20% decrease in body fat mass, a 9.5-cm decrease in waist circumference, and a 0.76-percentage-point decrease in glycated hemoglobin (HbA1c) (132). The findings of this trial and of others certainly suggest that at least as much emphasis should be placed on obesity and metabolic diseases as on muscle diseases in considering the potential indications for MSTN inhibition.

## MYOSTATIN AS A SKELETAL MUSCLE CHALONE

Based on what we now know about its expression, biological function, and mechanism of action, MSTN appears to have all of the salient properties of a chalone for skeletal muscle. MSTN is made specifically by myofibers, circulates in the blood, and acts back on myofibers to suppress growth. Superimposed on this signaling system is a complex regulatory network of multiple inhibitory binding proteins as well as other related ligands, such as activin A and BMPs, signaling through shared receptor components. This complexity likely reflects the systemic and local adaptations that muscle tissue must undergo in response to a wide range of physiological states and stimuli, such as food availability, temperature fluctuations, hormones, exercise, and injury. Although additional studies will be required to more precisely understand the roles that this pathway plays in various physiological states and the underlying mechanisms involved, it seems likely that skeletal muscle mass is subject to negative feedback control, with MSTN being the key mediator, and that the chalone hypothesis for the control of tissue mass may be apt in the case of skeletal muscle. One slight twist in the case of skeletal muscle is that MSTN appears to control myofiber growth directly rather than mitotic activity of muscle cells, as was originally proposed for how chalones might act to control tissue size (138).

The discovery of MSTN and its mechanism of action raises the question as to whether this type of negative feedback control may also operate in other tissues. In this regard, this same signaling pathway has been implicated as playing a negative role in the growth of other tissues besides skeletal muscle. One such tissue is bone, in which manipulation of this signaling pathway can lead to profound changes in density and mass. In particular, systemic treatment of mice with decoy forms of either ACVR2 (139) or ACVR2B (105, 140-144) or with an FST-based biologic (145) has been shown to cause significant and rapid increases in bone density. At least part of this effect results from inhibition of signaling directly to osteoblasts, as genetically targeting these type 2 receptors in osteoblasts can also lead to increased bone mass (146). The most dramatic effects have been seen upon simultaneously targeting the two type 1 receptors, ALK4 and ALK5, in osteoblasts, which leads to approximately tenfold increases in parameters such as bone volume and bone density (73). Some increases in bone density are also seen upon targeting *Mstn* and *Inhba*, but these effects are much milder than those seen upon targeting *Alk4* and *Alk5*, implying that other ligands are also likely involved. Clearly, much more work will be required to determine the identities of these ligands and their tissue sources as well as to elucidate the cellular responses to signaling. Nevertheless, the massive increases in bone mass seen upon targeting this pathway in osteoblasts are reminiscent of the massive increases in skeletal muscle mass seen upon targeting this pathway in myofibers, raising the possibility that this pathway may generally serve as a means for negative feedback regulation of tissue mass for not only muscle but perhaps other tissues as well.

## Figures and Tables

**Table 1:** Myostatin (MSTN) inhibitors tested or being tested in clinical trials

Drug	Company	Type	Indication	Phase	Reference
MYO-029	Wyeth/Pfizer	Anti-MSTN monoclonal antibody	Becker muscular dystrophy	Phase 1/2	120
			Facioscapulohumeral muscular dystrophy		
			Limb-girdle muscular dystrophy		
Domagrozumab	Pfizer	Anti-MSTN monoclonal antibody	Duchenne muscular dystrophy	Phase 2	121
			Limb-girdle muscular dystrophy	Phase 1/2	None
Landogrozumab	Eli Lilly	Anti-MSTN monoclonal antibody	Pancreatic cancer	Phase 2	130
			Hip replacement	Phase 2	128
			Muscle weakness following falls	Phase 2	129
REGN1033	Regeneron	Anti-MSTN monoclonal antibody	Sarcopenia	Phase 2	None
PINTA-745	Amgen/Atara	Anti-MSTN peptibody	End-stage renal disease	Phase 1/2	None
Taldefgrobep alfa	BMS/Roche	Anti-MSTN adnectin	Duchenne muscular dystrophy	Phase 2	None
	Biohaven		Spinal muscular atrophy	Phase 3	None
Apitegromab	Scholar Rock	Antipropeptide monoclonal antibody	Spinal muscular atrophy	Phase 3	None
RO7204239	Roche/Chugai	Antipropeptide monoclonal antibody	Spinal muscular atrophy	Phase 2/3	None
Bimagrumab	Novartis	Antireceptor monoclonal antibody	Sporadic inclusion body myositis	Phase 2/3	123-125
			Lung or pancreatic cancer	Phase 2	None
			Chronic obstructive pulmonary disease	Phase 2	131
			Sarcopenia	Phase 2	126, 127
			Hip fracture surgery	Phase 2	None
			Obesity with type 2 diabetes	Phase 2	132
ACE-031	Acceleron	Decoy receptor	Duchenne muscular dystrophy	Phase 2	122
